# Analysis of blood utilisation efficiency driven by clinical management and hospital heterogeneity

**DOI:** 10.3389/fpubh.2025.1668449

**Published:** 2025-09-25

**Authors:** Feng Wang, Zhi-guo Xu, Ke Lv, Jingxian Fei, Haiying Yang, Zequn Ou, Yun Song, Jingxian Song, Yizhu Chen, Le Wang

**Affiliations:** ^1^Huzhou Central Blood Station, Huzhou, China; ^2^Huzhou College of Life and Health, Huzhou, China

**Keywords:** discharged patients, per capita blood use, policy implementation linkage model, regional control of blood resources, patient blood management

## Abstract

**Background:**

Blood is a critical yet scarce medical resource, and improving the efficiency with which it is utilised remains a major global challenge. In 2019, China introduced Quality Control Indicators for Clinical Blood Use in an attempt to standardise management. However, significant discrepancies remain between the intended policy and its practical implementation, resulting in inefficiencies and safety concerns.

**Objective:**

This study aims to quantitatively evaluate the impact of administrative policies on clinical blood use, identify the main factors affecting the efficiency with which blood is utilised, and analyse how hospital level and type influence transfusion practices.

**Methods:**

A retrospective, multicentre study was conducted using data from 24 secondary and tertiary hospitals in Huzhou between 2020 and 2024. Key quality control indicators and a 25-point transfusion record scoring system were employed. Trends were analysed using ANOVA and chi-square tests, and hospital stratification was analysed using MANOVA. Predictors of blood use per discharged patient were identified using multiple linear regression and linear mixed-effects models.

**Results:**

Over 5 years, the number of transfusion technicians increased by 72%, transfusion record scores improved by 34.6%, and per capita blood use decreased by 46.9%. However, blood use in low-complexity surgeries increased by an abnormal 200%. Tertiary hospitals showed higher blood use but better documentation than secondary hospitals. Regression analysis revealed that technician density (*β* = −0.280) and transfusion record score (*β* = −0.202) were negatively associated with blood use, whereas surgical complexity was positively associated with it. Hospital grade and type also significantly influenced outcomes.

**Conclusion:**

Efficiency in blood utilisation is more strongly influenced by process standardisation and human resources than by hospital level or type alone. Rather than rigid indicators, policy incentives should emphasise precision transfusion strategies and dynamic quality management to align resource use with clinical need and support sustainable blood management systems.

## Introduction

As a non-renewable strategic reserve for saving lives, blood resources are increasingly becoming a serious challenge to the global public health system in terms of supply and demand tension and utilisation efficiency. To address this challenge, China promulgated the Indicators for Quality Control (QC) of Clinical Blood Use in 2019, aiming to improve the effectiveness of blood resource utilisation through standardised management. However, the policy has encountered significant resistance at the grassroots level—insufficient compliance and uneven dissemination of key technologies have led to a worrying “disconnect” between the policy vision and clinical practice. This disconnect is not only reflected at the level of specific operation (1–4), but also reflects systemic issues such as resource allocation, capacity building, and incentives at a deeper level. The serious consequences cannot be ignored: inefficient blood utilisation, increased potential safety risks of blood transfusion, and waste of valuable healthcare resources may ultimately affect patient outcomes and increase the burden on the system. As shown by Owusu-Ofori et al. (1) and studies in countries such as India (2), inconsistent guidelines, fragmented delivery systems, and lack of knowledge and training of healthcare professionals on current policies (3, 4) are common factors contributing to the policy-practice divide globally. At the same time, the rapid evolution of healthcare needs has placed greater demands on the timeliness and adaptability of policies (5, 6). Therefore, a central scientific question needs to be answered: how to effectively quantify and deconstruct the key drivers and mechanisms affecting the effectiveness of clinical blood use policy implementation, so as to bridge the gap between policy regulation and clinical practice?

To explore this issue in depth, this study focuses on the core QC indicator of ‘per capita blood use of discharged patients’. We systematically integrated the clinical blood use data of 24 secondary and above hospitals in the city from 2020 to 2024 with the QC centre’s supervisory and assessment records, constructing a “policy-implementation-impact” linkage assessment model through long-term, multi-centre retrospective analysis. This study aims to: (1) quantitatively monitor the actual impact of administrative regulatory measures on the implementation of clinical blood use specifications, and (2) provide an in-depth analysis of the underlying mechanisms that lead to changes in clinical blood use practices. The results of this study will provide solid quantitative evidence to inform the scientific evaluation of regulatory effectiveness, the precise identification of intervention targets and the optimisation of blood safety management strategies. Ultimately, this will serve the major goal of enhancing the efficiency of blood resource utilisation, ensuring patient safety and promoting the sustainable development of the national blood protection system.

## Research methods

### Data collection

The annual QC reports of all secondary and above hospitals (*n* = 24) in Huzhou City from 2020 to 2024 were extracted. The QC indexes and calculation formulas are shown in Table 1. The statistical values of the three indexes, “blood type review rate of blood recipient specimens,” “indoor QC rate of blood transfusion compatibility testing program” and “participation rate of inter-room quality assessment program of blood transfusion compatibility testing,” are at or close to 100% for each clinical blood hospital. As these three indicators reached or were close to 100% in each clinical blood hospital, they were excluded from the statistical analysis.

**Table 1:** Clinical blood QC indicators.

No.	Indicator name	Calculation formula
1	Number of blood transfusion professionals and technicians per 1,000 units	Number of full-time professionals and technicians in blood transfusion department / (Total number of units of blood used in medical institutions / 1,000)
2	Qualified rate of Clinical Blood Transfusion Application Forms	(Number of application forms filled out in a standardised manner and in compliance with the conditions of blood use / Total number of application forms received during the same period) × 100%
3	Recipient specimen blood type review rate	(number of recipient blood specimens reviewed for blood type / total number of recipient blood specimens received in the same period) × 100%
4	Indoor QC rate of transfusion compatibility testing programs	(number of transfusion compatibility testing programs for which indoor QC is conducted / total number of transfusion compatibility testing programs conducted by medical institutions) × 100%
5	Participation rate of inter-room quality assessment of transfusion compatibility testing programs	(number of transfusion compatibility testing programs participating in inter-room quality assessment / total number of transfusion compatibility testing programs of the participating inter-room quality assessment institutions) × 100%
6	Number of reported cases of adverse transfusion reactions per 1,000 blood transfusion person-times	Number of reported cases of adverse transfusion reactions / (Number of blood transfusion person-times / 1,000)
7	Average blood consumption for first and second level surgeries	Total number of units of blood used for first and second level surgeries / total number of first and second level surgeries in the same period
8	Average blood consumption for third and fourth level surgeries	Total number of units of blood used for third and fourth level surgeries / total number of third and fourth level surgeries in the same period
9	Autologous blood transfusion rate of surgical patients	(total number of units of autologous blood transfusion of surgical patients / (number of units of allogeneic blood transfusion of surgical patients + number of units of autologous blood transfusion of surgical patients in the same period)) × 100%.
10	Per capita blood use of discharged patients	Total units of blood used by discharged patients / Number of discharged patients in the same period

Scoring of blood transfusion medical records, 25-point scale (Table 2) was designed based on the Clinical Blood Transfusion Specifications and a double-blind evaluation by an expert group from the QC Centre (intra-group correlation coefficient ICC = 0.85). The annual score for each hospital was calculated as the mean value of six medical records.

**Table 2:** Blood transfusion quality evaluation scale (25-point system).

Assessment criteria	Project evaluation	Evaluation standard	Score
Rationalisation of blood transfusion (10 points)	1. Red blood cell transfusion	Reasonable: surgical Hb < 70 g/L or medical Hb < 60 g/L with symptoms Unreasonable: Hb > 100 g/L without active bleeding Partially reasonable: in-between Deduct points where appropriate	0–10 points
	2. Plasma transfusion	Reasonable: PT/APTT >1.5x or coagulation factor deficiency Unreasonable: nutritional support, volume expansion, paired transfusion Partially reasonable: deduct points for other conditions as appropriate	0–10 points
	3. Platelet transfusion	Reasonable: surgical PLT < 50 × 10^9^/L or medical PLT < 10 × 10^9^/L (prophylaxis) Unreasonable: surgical PLT > 100 × 10^9^/L or medical PLT > 50 × 10^9^/L Partially reasonable: close to standardised discretionary points	0–10 points
The standardisation of medical records (10 points)	1. informed consent	Signature, risk information, blood transfusion varieties complete → 2 points Missing any one → 0.5 points / item Three missing → 2 points	0–2 points
	2. Pre-transfusion examination	Hepatitis B/HIV and other five items + blood type review complete → 2 points Missing any one → 1–2 points	0–2 points
	3. Blood transfusion process record	Complete record of start and stop time, speed, adverse reaction observation → 2 points Missing any one item → 0.5 points/item deducted	0–2 points
	4. Post-transfusion evaluation	Hb/PLT test 24–48 h post-infusion + efficacy comparison → 2 points Absence of test or no comparison → 1 point	0–2 points
	5. Adverse reaction treatment	Symptom records + measures + reporting complete → 2 points Missing any one → 1–2 points	0–2 points
Process Compliance (5 points)	1. Double checking	Double check the blood bag information and patient identity before transfusion → 3 points Failure to implement → 3 points	0/3 points
	2. Blood bag management	After transfusion, the blood bag is returned to the transfusion department + preservation ≥ 24 h → 1 point Not returned or insufficiently preserved→1 point	0/1 points
	3. Aseptic operation	The use of specialised blood transfusion device + not add drugs → 1 point Illegal operation → 1 point	0/1 points

### Trend analysis

The statistics cover blood transfusion medical record scores and the remaining seven QC indicators of clinical blood use in hospitals above level two in Huzhou City from 2019 to 2024. These include the number of blood transfusion professionals and technicians per 1,000 units of blood used and the qualification rate of clinical blood transfusion application forms., the number of adverse transfusion reaction cases reported per 1,000 transfusions, the average amount of blood used per first- and second-degree surgery, the average amount of blood used per third- and fourth-degree surgery, the autologous transfusion rate for surgical patients, and the average amount of blood used per patient discharged from hospital. We analysed the trend of each data set over the past 5 years.

### Stratified comparison

The 24 hospitals were classified according to hospital class and type as tertiary (11), secondary (13), general (15) and specialised (9) hospitals. The differences in QC index data and transfusion chart scores between the different classes and types of hospitals over a five-year period were analysed using multivariate analysis of variance (ANOVA).

### Multiple linear regression modelling

The dependent variable was “blood use per discharged patient” (Box-Cox transformed to satisfy normality), and the remaining six QC indicators were the independent variables. The control variables were hospital grade (tertiary = “0,” secondary = “1”), hospital type (specialty hospital = “0,” general hospital = “1”), and transfusion medical record score, which were inflated by variance. Multiple linear regression equations were constructed using stepwise regression (entry criteria *p* < 0.05, exclusion *p* > 0.10) and the variance inflation factor (VIF) test was used to remove the covariance (threshold VIF < 5). Residual independence was tested using the Durbin-Watson test (1.5 < DW < 2.5).

### Linear mixed effects model analysis

A linear mixed-effects model was used to account for clustering effects at hospital level, and a compound symmetric (CS) covariance structure was employed to model temporal correlations in data from different years within the same hospital.

### Statistical analysis

Categorical variables were compared using the chi-squared test (with Yates’s correction for expected frequencies of less than 5). Rates were compared using the chi-squared test. Continuous variables were compared using the Shapiro–Wilk normality test, followed by the independent samples t-test, the variance test (chi-squared test) or the Mann–Whitney U-test (for non-normal distributions). Model diagnosis was performed using residual normality (Q–Q plots) and heteroscedasticity (Breusch–Pagan test). Statistical software: SPSS 27.0 (statistical analysis, linear regression and model diagnosis) and GraphPad Prism 10.4 (trend visualisation).

## Results

### Trends in QC indicators and “transfusion history score”

**Figure 1:**
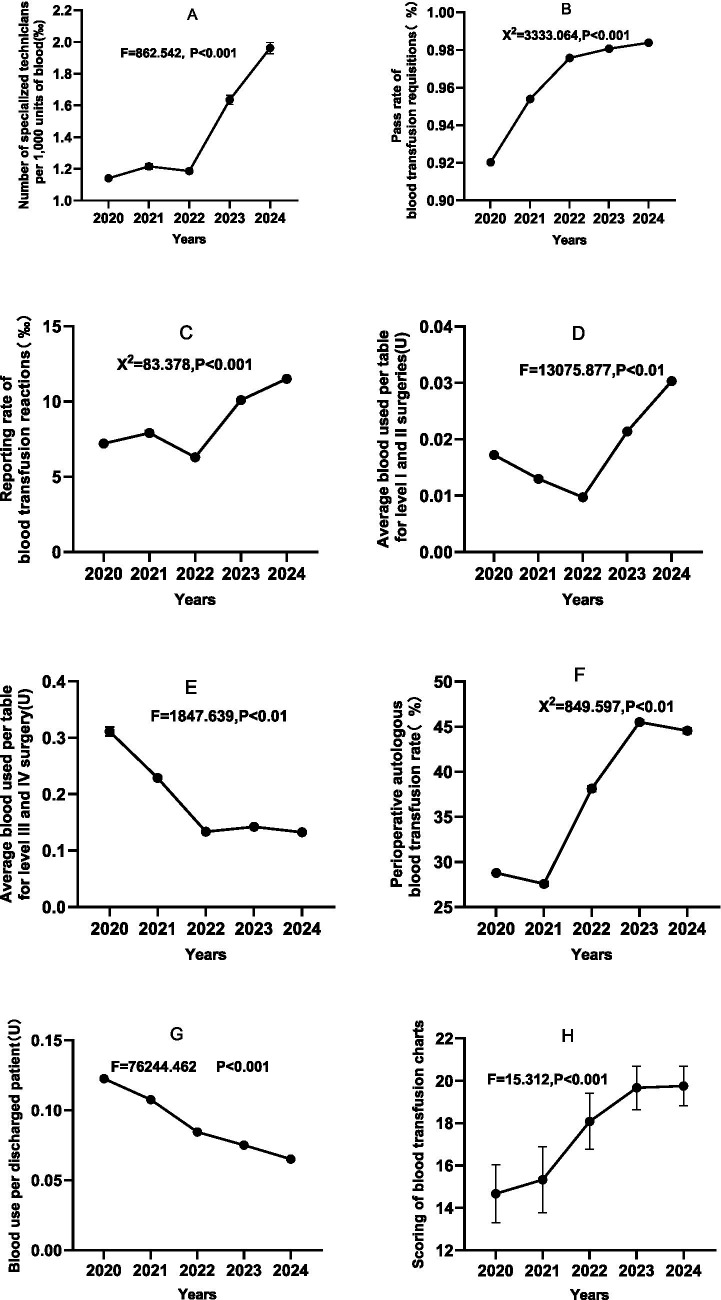
Trends in clinical blood QC indicators (**A**: Number of blood transfusion professionals and technicians per 1,000 units; **B**: Qualified rate of Clinical Blood Transfusion Application Forms; **C**: Number of reported cases of adverse transfusion reactions per 1,000 blood transfusion persontimes; **D**: Average blood consumption for first and second level surgeries; **E**:Average blood consumption for third and fourth level surgeries; **F**: Autologous blood transfusion rate of surgical patients; **G**: Per capita blood use of discharged patients) and transfusion history scores, 2020–2024.

A one-way analysis of variance (ANOVA) and a Pearson’s chi-square test revealed a significant upward trend in the number of specialised blood transfusion technicians per 1,000 units of blood used from 2020 to 2024 (1.14 → 1.96, an increase of 72%), peaking in 2024 (M = 1.96, 95% CI [1.93, 2.00]). One-way ANOVA also revealed a significant year effect (*F*(4, 469,098) = 862.54, *p* < 0.001). Transfusion requisition pass rates continued to improve (2020: 92.0% → 2024: 98.4%), and a chi-square test confirmed a significant year effect (χ^2^ = 3333.06, *p* < 0.001). The reporting rate of adverse transfusion reactions increased continuously (2020: 0.72% → 2024: 1.15%), rising by 59.7%, and was significantly higher from 2023 onwards (2023: 1.01, 95% CI [1.00, 1.02] to 2024: 1.15, 95% CI [1.14, 1.16]). A chi-squared test confirmed that the reporting rates in different years were qualitatively different (χ^2^ = 83.378, *p* < 0.001). There was a substantial increase in perioperative autotransfusion rates (2020: 28.8% → 2024: 44.6%), peaking at 45.5% in 2023 (95% CI [45.27, 45.73]). There was a significant increase between 2022 and 2023 (38.1% → 45.5%), followed by a small decrease in 2024 which was significant according to the chi-square test (χ^2^ = 849.60, *p* < 0.001). Regarding the efficiency of surgical blood use, the average blood usage per procedure in first- and second-level surgeries fluctuated (2020: 0.017 U → 2022: 0.010 U → 2024: 0.030 U), with a sudden increase in usage in 2023–2024 (0.021 U → 0.030 U). This was contrary to the overall optimisation trend, as shown by the analysis of variance (*F*(4, 580,880) = 13,630; 0.20, *p* < 0.001). The average blood usage per case for tertiary and quaternary surgeries continued to decrease significantly (2020, 0.311 U → 2024: 0.133 U, a 57.2% decrease), as revealed by a significant ANOVA (*F*(4, 254,818) = 425.73, *p* < 0.001). A significant trend was also observed over time in the ‘blood use per patient discharged and transfusion history score’, with *F*-values of 76,244.46 and 15.312, respectively (*p* < 0.001). The mean value of blood use per patient discharged decreased gradually from 0.123 units (95% CI, 0.122–0.123) in 2020 to 0.065 units (95% CI, 0.065–0.065) in 2024, representing a 46.9% decrease. Conversely, the mean transfusion medical record score increased from 14.67 (95% CI, 13.30–16.03) in 2020 to 19.75 (95% CI, 18.81–20.69) in 2024, indicating an improvement in the standardisation of transfusion practice in clinical blood hospitals. See Figures 1A–H.

### Stratified comparison

**Figure 2:**
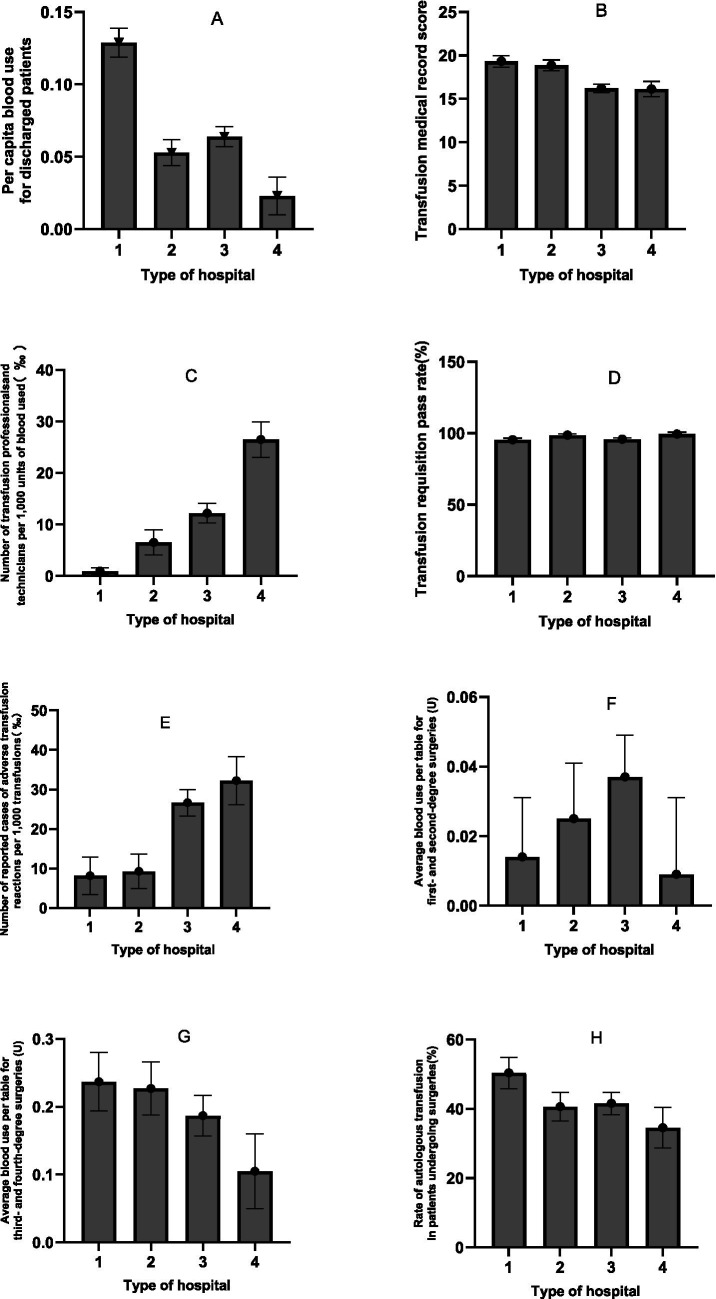
Means and standard deviations of indicators (**A**: Pre capita blood use for discharged patients; **B**: Transfusion medical record score; **C**: Number of transfusion professionals and technicians per 1,000 units of biood used(‰); **D**: Transfusion requisition pass rate; **E**: Number of reported cases of adverse transfusion reactions per 1,000 transfusions; **F**: Average blood use per table for first- and second-degree surgeries(U); **G**: Average blood use per table for third- and fourth-degree surgeries(U); **H**: Rate of autologous transfusion in patients undergoing surgeries) for different hospital grades and types.

A multivariate analysis of variance (MANOVA) was conducted to examine the effects of hospital grade (Level III vs. Level II) and hospital type (general vs. specialised) on eight transfusion-related performance indicators. The analysis incorporated 120 annual reports from 24 hospitals over a five-year period. The reports were stratified by hospital level (tertiary hospitals: *n* = 55; secondary hospitals: *n* = 65) and type (general hospitals: *n* = 75; specialty hospitals: *n* = 45). Key transfusion-related indicators are presented in Table 3 as means with 95% confidence intervals (CIs) and standard deviations (SDs; Figure 2).

1. Level III general hospitals.
2. Level III specialised hospitals.
3. Level II general hospitals.
4. Level II specialised hospitals.

**Table 3:** Multivariate tests^a^.

Effect	Value	F	Hypothesis df	Error df	Sig.
Intercept	0.997	4575.158^b^	8.000	109.000	0.000
Level	0.451	11.196^b^	8.000	109.000	0.000
Type	0.291	5.604^b^	8.000	109.000	0.000
Level * Type	0.027	1.474^b^	8.000	109.000	0.175

a Design: Intercept + Level + Type + Level * Hospital Type.

b Exact statistic.

Box’s M test (M = 739.426, *F* = 5.759, *p* < 0.001) permitted subsequent analyses to proceed, given that MANOVA is robust to chi-squaredness of the covariance matrix (especially when sample sizes between groups are similar). Multiple effects tests (Pillai’s trace prevailed) were significant for the main effects of hospital class (Pillai’s trace = 0.451, *F* = 11.196, *p* < 0.001, partial η^2^ = 0.451) and hospital type (Pillai’s trace = 0.291, *F* = 4.86, *p* < 0.001). The hospital class × hospital type interaction effect was not significant (Pillai’s Trace = 0.098, *F* = 1.474, *p* = 0.175; see Table 3).

The between-subjects effect test for tertiary vs. secondary hospitals showed the following results:

### Main effect of hospital grade

The results revealed a significant main effect of hospital grade on multiple indicators (Pillai’s trace; *p* < 0.05). Specifically:

- Per capita blood use for discharged patients was significantly higher in Level III hospitals than in Level II hospitals (*F*(1, 119) = 22.322, *p* < 0.001, partial η^2^ = 0.161).
- Transfusion medical record scores were significantly higher in Level III hospitals (*F*(1, 119) = 18.449, *p* < 0 0.001, partial η^2^ = 0.137).
- There was a significant difference in the number of transfusion professionals and technicians per 1,000 units of blood between grades (*F*(1, 119) = 33.733, *p* < 0 0.001, partial η^2^ = 0.225), with a higher mean count in Level II hospitals.
- The number of reported adverse transfusion reactions per 1,000 transfusions was also significantly higher in Level II hospitals (F(1, 119) = 18.972, *p* < 0 0.001, partial η^2^ = 0.141).
- The average amount of blood used per case for third- and fourth-degree surgeries was also significantly influenced by grade (F(1, 119) = 4.069, *p* = 0 0.046, partial η^2^ = 0.034), with level III hospitals using more blood.
- However, no significant main effect of grade was found for the transfusion requisition pass rate (*p* = 0 0.608), blood use in minor surgeries (*p* = 0 0.847) or the autologous transfusion rate (*p* = 0.104).

### Main effect of hospital type

A significant main effect of hospital type was also observed.

- Per capita blood use for discharged patients was significantly higher in general hospitals than in specialised hospitals (*F*(1, 119) = 34.280, *p* < 0.001, partial η^2^ = 0.228).
- There was a significant difference in the number of transfusion professionals and technicians per 1,000 units of blood between types (F(1, 119) = 13.703, *p* < 0.001, partial η^2^ = 0.106), with specialised hospitals showing a higher mean count.
- The transfusion requisition pass rate was also significantly higher in specialised hospitals (F(1, 119) = 9.662, *p* = 0.002, partial η^2^ = 0.077).
- However, hospital type did not have a significant effect on transfusion record scores (*p* = 0 0.692), adverse reaction reports (*p* = 0.482), blood use for any surgical grade (*p* > 0.05) or the autologous transfusion rate (*p* = 0.067).

### Interaction effect (grade × type)

The interaction effect between hospital grade and type was not statistically significant for any of the eight dependent variables (all *p* > 0.05). This indicates that the effect of hospital grade on these indicators is consistent across different hospital types, and vice versa.

### Effect size

The effect sizes (partial eta squared) for the significant findings ranged from small to medium (0.034 to 0.228). The largest effect was observed for the impact of hospital type on per capita blood use (partial eta squared = 0.228), as shown in Table 4.

- R Squared = 0.310 (Adjusted R Squared = 0.293)
- R Squared = 0.157 (Adjusted R Squared = 0.135)
- R Squared = 0.243 (Adjusted R Squared = 0.224)
- R Squared = 0.074 (Adjusted R Squared = 0 0.050)
- R Squared = 0.147 (Adjusted R Squared = 0.125)
- R Squared = 0.015 (Adjusted R Squared = −0.010)
- R Squared = 0.036 (Adjusted R Squared = 0.011)
- R Squared = 0.050 (Adjusted R Squared = 0.026)

**Table 4:** Tests of between-subjects effects.

Source	Dependent variable	df	Mean square	F	Sig.	Partial eta squared
Level	Per capita blood use for discharged patients	1	0.056	22.322	0.000	0.161
	Transfusion medical record score	1	212.674	18.449	0.000	0.137
	Number of transfusion professionals and technicians per 1,000 units of blood used (‰)	1	6080.989	33.733	0.000	0.225
	Transfusion requisition pass rate	1	8.106	0.265	0.608	0.002
	Number of reported cases of adverse transfusion reactions per 1,000 transfusions	1	10733.392	18.972	0.000	0.141
	Average blood use per table for first- and second-degree surgeries (U)	1	0.000	0.038	0.847	0.000
	Average blood use per table for third- and fourth-degree surgeries (U)	1	0.187	4.069	0.046	0.034
	Rate of autologous transfusion in patients undergoing surgeries	1	2620.310	2.692	0.104	0.024
Type	Per capita blood use for discharged patients	1	0.086	34.280	0.000	0.228
	Transfusion medical record score	1	1.823	0.158	0.692	0.001
	Number of transfusion professionals and technicians per 1,000 units of blood used (‰)	1	1387.982	13.703	0.000	0.106
	Transfusion requisition pass rate	1	295.618	9.662	0.002	0.077
	Number of reported cases of adverse transfusion reactions per 1,000 transfusions	1	281.308	0.497	0.482	0.004
	Average blood use per table for first- and second-degree surgeries (U)	1	0.002	0.223	0.638	0.002
	Average blood use per table for third- and fourth-degree surgeries (U)	1	0.054	1.170	0.282	0.010
	Rate of autologous transfusion in patients undergoing surgeries	1	1762.417	3.418	0.067	0.029
Level * Type	Per capita blood use for discharged patients	1	0.008	3.128	0.080	0.026
	Transfusion medical record score	1	0.840	0.073	0.788	0.001
	Number of transfusion professionals and technicians per 1,000 units of blood used (‰)	1	477.461	2.649	0.106	0.022
	Transfusion requisition pass rate	1	0.353	0.012	0.915	0.000
	Number of reported cases of adverse transfusion reactions per 1,000 transfusions	1	123.719	0.219	0.641	0.002
	Average blood use per table for first- and second-degree surgeries (U)	1	0.009	1.236	0.269	0.011
	Average blood use per table for third- and fourth-degree surgeries (U)	1	0.033	0.712	0.400	0.006
	Rate of autologous transfusion in patients undergoing surgeries	1	46.213	0.090	0.765	0.001

### Multiple linear regression modelling

The model constructed using stepwise regression was statistically significant (*F* = 15.067, *p* < 0.001) and explained 39.2% of the variation in blood usage per patient discharged from hospital (adjusted R^2^ = 0.392). The model residuals were independent (Durbin-Watson statistic = 2.009) and there were no serious multicollinearity issues between the predictor variables (VIF < 2.23). Hospital type was the primary negative predictor (standardised *β* = −0.457), with a significant increase in per capita blood use of 0.056 units in general hospitals compared to specialty hospitals (95% CI: −0.077 to −0.035, t(114) = −5.261, *p* < 0.001). Technician staffing (standardised *β* = −0.295) and transfusion history score (standardised *β* = −0.212) synergistically suppressed blood use: an increase of 1 technician per 1,000 units of blood used decreased per capita blood use by 0.001 units (*p* = 0.001), and each 1-point improvement in the transfusion history score decreased blood use by 0.003 units (*p* = 0.021). Surgical complexity was also a significant factor, with per capita blood use rising by 0.018 units for every 1-unit increase in blood use for grades 3 and 4 surgeries (*β* = 0.226, *p* = 0.003), which supports the idea of a rigid demand for high-difficulty surgeries. Conversely, an increase in hospital grade (low grade to high grade) decreased blood use (*β* = −0.343, *p* = 0.002), suggesting that high-grade hospitals offset the effect of surgical complexity through effective management (Table 5). The standardised regression coefficients (β) and significance of each predictor variable are shown in Table 6. The final regression equation is as follows:

**Table 5:** Results of multiple linear regression analysis of blood use per patient discharged from the hospital (*n* = 120).

Dependent variable	B(95%CI)	SE	Beta	t	P	VIF
(Constant)	0.170(0.116–0.224)	0.027		6.235	0.000	
Number of transfusion professionals and technicians per 1,000 units of blood used (‰)	−0.001(−0.002–−0.001)	0.000	−0.280	−3.492	0.000	1.299
Type	−0.049(−0.068–−0.029)	0.010	−0.396	−5.043	0.000	1.247
Level	−0.042(−0.063–−0.021)	0.011	−0.353	−3.881	0.003	1.672
Transfusion medical record score	−0.003(−0.006–−0.001)	0.001	−0.202	−2.583	0.011	1.239
Average blood use per table for third- and fourth-degree surgeries(U)	0.046(0.006–0.087)	0.020	0.168	2.299	0.023	1.084

Adjusted R^2^ = 0.410, F = 17.557, *p* < 0.001, DW = 2.001.

**Table 6:** Factors associated with *per capita* blood use for discharged patients: a linear mixed-effects model analysis.

Parameter	Estimate	Std. error	df	t	*p*	95% CI
Fixed Effects						
Intercept	0.023	0.019	24.000	1.187	0.247	(−0.017, 0.063)
Hospital Level (Level III vs. II)	0.030	0.024	24.000	1.249	0.224	(−0.019, 0.079)
Hospital Type (Specialised vs. General)	0.041	0.022	24.000	1.855	0.076	(−0.005, 0.086)
Level × Type Interaction	0.035	0.030	24.000	1.182	0.249	(−0.026, 0.097)
Random Effects	Variance	Std. Error				
Between-Hospitals (Intercept)	≈ 0	—				
Repeated Measures (CS Structure)						
Residual Variance	0.00163	0.00024				
Within-Hospital Covariance	−0.00028	0.00033				

Blood use per patient discharged from hospital = 0.170–0.001 × number of transfusion professionals and technicians per 1,000 units of blood used − 0.049 × hospital grade − 0.042 × hospital grade − 0.003 × transfusion chart score + 0.046 × average blood use per surgical table for three or four levels of surgery.

### Linear mixed effects model analysis

The mixed-model results indicate that the variance estimate for the random intercept is close to zero and has been flagged as redundant (Variance ≈ 0), which suggests that there are no significant differences in baseline per capita blood usage among hospitals. The covariance parameters show that variability in the data is mainly due to fluctuations within hospitals over time (residual variance = 0.001625). Furthermore, none of the fixed effects (hospital grade, type, and their interaction) were significant (all *p* > 0.05). These findings align with those from the random effects analysis, confirming that macro-level hospital classification characteristics are ineffective predictors of per capita blood usage, as shown in Table 6.

## Discussion

This study uses multi-indicator trend analysis to reveal the synergistic effects of implementing blood management policies within regional healthcare systems. The analysis indicates that, over a five-year period, the number of blood transfusion technicians increased significantly by 72.0%. This change exhibits a clear temporal correlation with improvements in transfusion record quality (as measured by transfusion history scores), which increased by 34.6%. This finding corroborates the view proposed by Naveen Bansal et al. that ‘professional staffing forms the foundation of transfusion safety’ (7–9). Furthermore, multivariate regression analysis indicates that technician density is a significant negative predictor of per capita blood consumption (*β* = −0.280, *p* < 0.001). This suggests that investments in human capital and process standardisation are more critical policy drivers than administrative oversight alone.

Concurrently, the volume of red blood cell transfusions per capita decreased by 46.9%, equivalent to approximately 123,000 unnecessary transfusions being avoided each year. The compliance rate for completed transfusion request forms increased to 98.4%, and the rate of adverse event reports rose by 59.7%, indicating strengthened pre-approval review and post-event feedback mechanisms.

However, an abnormal increase in blood usage was observed in Classes I and II surgeries (from 0.010 U in 2022 to 0.030 U in 2024, representing a 200% increase; *F* = 13,630.20, *p* < 0.001). However, multivariate analysis of variance revealed no significant differences in blood usage across hospital types/grades for these procedures (*p* > 0.05). This suggests a potential widespread misuse of transfusion indications in low-risk surgeries. Autologous transfusion rates notably peaked in 2023 at 45.5% (95% CI [45.27, 45.73]), which coincided with the rise in blood usage for Class I and II surgeries. However, this increase in autologous transfusion rates did not improve overall blood utilisation efficiency (linear regression *p* = 0.299). Further analysis revealed no significant differences in perioperative autologous transfusion rates among hospitals (*F* = 3.418 for level and *F* = 2.032 for type; *p* > 0.05), which contradicts the expectation that higher-level hospitals would have higher rates due to a greater proportion of complex surgeries.

The aforementioned anomalous results may be related to the design of the assessment mechanism. Although autologous blood transfusion can reduce the risks associated with allogeneic transfusion (26, 27), when the implementation rate is used as a rigid assessment indicator, it can encourage institutions to collect autologous blood for non-indicated purposes during low-risk surgeries. This results in the misallocation of resources (e.g., resources needed for high-risk surgeries being diverted) (10–13). In the regression model, medical record scores showed a negative correlation (B = −0.003), with tertiary hospitals scoring significantly higher than secondary hospitals. This suggests that lower-level hospitals could improve their blood management systems, reflecting the potential ‘double-edged sword’ effect of policy incentives on clinical practice (14–16).

Therefore, evidence-based autotransfusion guidelines referencing the AABB Perioperative Autologous Blood Collection and Transfusion Standard (17) are necessary. The assessment system should shift from evaluating implementation rates alone to evaluating both the implementation rate and the indication compliance rate, while promoting a ‘personalised threshold strategy’ (18). For example, autologous blood collection should be restricted to low-risk procedures (e.g., Grade I hernia repair) with a restrictive transfusion threshold (Hb < 7 g/dL). Conversely, for moderate-to-high-risk surgeries (e.g., radical tumour resection), multimodal blood management plans should be tailored based on factors such as preoperative anaemia status and surgical scope. This approach ensures precise alignment between resource allocation and clinical needs, thus optimising academic transfusion practices.

Tertiary general hospitals exhibited the typical coexistence of ‘high blood usage and high quality control’, demonstrating the highest per capita blood usage (M = 0.129 U) and optimal transfusion record scores (19.32 points). Multivariate linear regression analysis indicates that, although increased surgical complexity leads to higher blood demand, a higher hospital tier is significantly associated with reduced per capita blood usage (*β* = −0.353, *p* = 0.003). However, mixed-effects modelling revealed a more complex mechanism. As shown in Table 6, variations in blood usage were driven less by inherent attributes such as hospital grade or type and more by dynamic factors over time, such as the implementation of short-term clinical quality improvement initiatives. This suggests that future research on blood utilisation efficiency should focus on tracking and evaluating process-oriented management measures rather than merely comparing outcome metrics across different hospital categories.

Lasocki et al. (19) demonstrated that implementing standardised PBM significantly reduced red blood cell transfusion rates by 28% (*p* < 0.001). Mitchell’s team further validated the efficacy of PBM in high-complexity orthopaedic surgeries, demonstrating that adopting a restrictive transfusion strategy (haemoglobin threshold <8 g/dL) reduced blood usage by 32% in Level III and IV surgeries without increasing the risk of complications (*p* = 0.03) (20). Furthermore, multiple pieces of clinical evidence indicate that standardised practice models are essential for achieving precision transfusion. In specialised fields such as transplantation, oncology and sickle cell disease, compliance with standardised transfusion protocols is between 50 and 75% (21). In contrast, general hospitals (particularly orthopaedic and community hospitals) still exhibit significant heterogeneity between hospitals in transfusion indication management and process control (22).

This study also found that the overall per capita blood usage in specialist hospitals was significantly lower than in general hospitals (one-way ANOVA, *F* = 34.280, *p* < 0.001). This finding further supports the positive role of precision transfusion strategies in enhancing blood utilisation efficiency.

This study has several limitations. Firstly, the analysis did not adjust for patient-specific complexity or case mix variation [e.g., by using measures such as Diagnosis-Related Groups (DRGs) or Case Mix Index (CMI)] (23). This implies that the observed differences in blood usage between hospitals of different levels and types may be due to disparities in patient disease severity and surgical complexity rather than differences in management efficiency or a combination of both. Therefore, the results reported in this study should be interpreted as unadjusted observational differences. Although the proportion of high-complexity surgeries was included as a proxy for complexity, future studies should incorporate patient-level data to more accurately distinguish the independent contributions of case-mix characteristics and management practices to disparities in blood usage (24, 25).

Secondly, empirical evidence from China’s urban context suggests that policy interventions focused on strengthening technical staffing and standardising processes may significantly improve blood utilisation efficiency and safety culture levels. However, the generalisability of these findings may be influenced by patient demographics, hospital organisational structures, and prevailing governance models in target regions. Therefore, while core principles such as strengthening human resource development, standardising operational procedures and reforming incentive mechanisms are universally relevant, the design and implementation of specific policies require adaptation to local conditions.

In summary, this study further highlights that the future focus of blood management should shift from simple metrics to a patient-centred, data-driven, intelligent framework. This encompasses performance evaluation with data-based risk adjustment, widespread adoption of validated Patient Blood Management (PBM) protocols across all surgical specialties and incentive policies that emphasise clinical outcomes and transfusion rationality rather than quantitative indicators alone. By implementing these measures, healthcare systems can work together to improve the quality and efficiency of transfusion practices.

## Data Availability

The original contributions presented in the study are included in the article/supplementary material, further inquiries can be directed to the corresponding author.
